# Comprehensive analysis of competitive endogenous RNAs network associated with head and neck squamous cell carcinoma

**DOI:** 10.1038/s41598-018-28957-y

**Published:** 2018-07-12

**Authors:** Xiao-Nan Fang, Miao Yin, Hua Li, Cheng Liang, Cong Xu, Gui-Wen Yang, Hua-Xiang Zhang

**Affiliations:** 1 Shandong Provincial Key Laboratory of Animal Resistance Biology, College of Life Sciences, Shandong Normal University, Jinan, 250014 China; 2 Key Laboratory of TCM Data Cloud Service in Universities of Shandong, School of Information Engineering, Shandong Management University, Jinan, 250357 China; 3 School of Information Science & Engineering, Shandong Normal University, Jinan, 250358 China; 4 College of Pharmacy, Binzhou Medical University, Yantai, 264003 China

## Abstract

Long non-coding RNAs (lncRNAs) can regulate gene expression directly or indirectly through interacting with microRNAs (miRNAs). However, the role of differentially expressed mRNAs, lncRNAs and miRNAs, and especially their related competitive endogenous RNAs (ceRNA) network in head and neck squamous cell carcinoma (HNSCC), is not fully comprehended. In this paper, the lncRNA, miRNA, and mRNA expression profiles of 546 HNSCC patients, including 502 tumor and 44 adjacent non-tumor tissues, from The Cancer Genome Atlas (TCGA) were analyzed. 82 miRNAs, 1197 mRNAs and 1041 lncRNAs were found to be differentially expressed in HNSCC samples (fold change ≥ 2; *P* < 0.01). Further bioinformatics analysis was performed to construct a lncRNA-miRNA-mRNA ceRNA network of HNSCC, which includes 8 miRNAs, 71 lncRNAs and 16 mRNAs. Through survival analysis based on the expression profiles of RNAs in the ceRNA network, we detected 1 mRNA, 1 miRNA and 13 lncRNA to have a significant impact on the overall survival of HNSCC patients (*P* < 0.05). Finally, some lncRNAs, which are more important for survival, were also predicted. Our research provides data to further understand the molecular mechanisms implicated in HNSCC.

## Introduction

Head and neck squamous cell carcinoma (HNSCC) is the sixth most commonly reported malignancy worldwide^1^. There are about 500,000 new HNSCC patients diagnosed worldwide each year, and the death rate of new cases is about 20%^2,3^. Although comprehensive treatment, including surgical resection, radiotherapy, and chemotherapy, continues to develop, the overall 5-year survival rate of HNSCC patients has not been significantly improved over the past few decades and has not exceeded 50%. Thus, HNSCC is considered a malignant tumor with low survival rate^4^. Therefore, understanding the molecular nature of HNSCC carcinogenesis is a pivotal step for meliorating early diagnosis, predicting prognosis, and developing effective therapeutics^5^.

In recent years, the regulatory network composed of long non-coding RNAs (lncRNAs), microRNAs (miRNAs) and messenger RNAs (mRNAs) has gained a great interest in the study of molecular biological mechanisms involved in the process of tumor occurrence and progression. lncRNA refers to a non-coding RNA transcript with a length greater than 200 nucleotides, which is located in the nucleus and cytoplasm of eukaryotic cells^6^. A large number of clinical and experimental studies have shown that some lncRNAs play important roles in the emergence and development of malignant tumors^7^.

miRNA is an endogenous single stranded RNA molecule of 22 nucleotides length that does not encode for a protein. miRNA inhibits the expression of a target gene by complementation binding of its seed region to the microRNA response elements (MREs) on the mRNA^8^. The regulation network between miRNA and target genes is involved in a variety of biological processes, including tumor occurrence and metastasis^9,10^. In recent years, research on the role of miRNAs and their implication in the regulation of initiation and development of HNSCC has advanced, and a large number of papers have been published. In 2016, Irani provided a comprehensive literature review of the role of miRNAs in head and neck cancer metastasis^11^. These studies have laid a good foundation for understanding the mechanism of lncRNA/miRNA/mRNA interactions.

In 2011, Salmena and colleagues presented the competing endogenous RNA (ceRNA) hypothesis which states that the pool of mRNAs, lncRNAs, and other non-coding RNAs share common MREs with miRNAs, which can act as natural miRNA “sponges” and inhibit miRNA function through competitive binding to MREs on the target mRNA^12^. This theory is based on the fact that lncRNAs can regulate expression of target regulatory genes not only by direct interaction, but also indirectly through competitive binding with miRNAs, as they contain MREs which can bind to the core seed sequence of miRNA. At present, the ceRNA regulation theory has been proven to be implicated in the development of cancer by some related studies. For example, Guo *et al*. found that lncRNA-*BGL3* was a target of *miR-17*, *miR-93*, *miR-20a*, *miR-20b*, *miR-106a* and *miR-106b*, microRNAs that repress mRNA of phosphatase and tensin homolog (*PTEN*). Further experiments showed that lncRNA-*BGL3* operated as a competitive endogenous RNA for binding these microRNAs to cross-regulate *PTEN* expression^13^. The lncRNA gene, *HLUC*, acts as a ceRNA of *PRKACB* in liver cancer by competing with *miR-372*, thereby reducing *miR-372*’s inhibitory effect on CREB and causing up-regulation of CREB^14^. With the development of bioinformatics technology, more researchers have adopted data analysis and mining methods to study ceRNA networks. In addition, some representative databases, such as miRTarBase^15^, miRDB^16^, TargetScan^17^ and StarBase^18^ provide data and useful resources for studying ceRNA networks. The Cancer Genome Atlas (TCGA) research team has published a comprehensive resource for multidimensional molecular spectroscopy that stores a large number of tumor samples. These datasets make it feasible to build a ceRNA regulatory network in cancer.

In recent years, numerous studies have confirmed that the lncRNA-miRNA-mRNA ceRNA regulatory network is implicated in the development of gastric, liver, breast, pancreatic and bladder cancer^19–23^. However, similar studies on HNSCC are limited, and there is still a lack of comprehensive analysis of lncRNAs and miRNAs related to HNSCC based on high-throughput sequencing and large-scale sample size. In this paper, we obtained RNA expression data and compared the expression profiles between 44 normal tissues and 502 tumor tissues of HNSCC. Following, we identified differentially expressed mRNAs, miRNAs and lncRNAs between the samples from HNSCC patients. Finally, 8 miRNAs, 16 mRNAs and 71 lncRNAs were selected to build the lncRNA-miRNA-mRNA ceRNA network. Based on the survival analysis of RNAs in the ceRNA network, we analyzed the lncRNAs that significantly affect the survival and prognosis of HNSCC patients.

## Materials and Methods

### Patients and TCGA data retrieval

The RNA sequence data of 546 samples with head and neck squamous cell carcinoma were retrieved from the TCGA data portal. The TCGA dataset (https://portal.gdc.cancer.gov/), being comprised of more than two petabytes of genomic data, is publically available, and this genomic information helps the cancer research community to improve the prevention, diagnosis, and treatment of cancer. This study is in accordance with the publication guidelines provided by TCGA (https://cancergenome.nih.gov/publications/publicationguidelines). Since the data comes from the TCGA database, no further approval was required from the Ethics Committee.

### RNA sequence data processing

The RNA expression data (level 3) of 546 HNSCC samples were downloaded from the TCGA data portal (up to Aug 29, 2017). The RNA and miRNA sequence data from the 546 samples had been derived from the IlluminaHiSeq_RNASeq and the IlluminaHiSeq_miRNASeq sequencing platforms; all the data were freely available to download. The sequencing data of the 546 samples contained the corresponding RNA-seq and miRNA-seq data. 546 samples were divided into 2 cohorts: 502 tumor samples and 44 normal samples.

In this paper, we mainly used the program code written in Perl and R language to analyze and deal with RNA data.

### Identification of differentially expressed mRNAs (DEmRNAs), miRNAs (DEmiRNAs) and long non-coding RNAs (DEIncRNAs)

We identified mRNAs and lncRNAs by using the Ensembl database (http://www.ensembl.org/index.html, version 89)^24^. In this study, lncRNAs and mRNAs that were not included in the database were excluded.

Before conducting differential expression analysis, we wrote the R language code to ensure that all unexpressed RNAs was filtered out. This was carried out by deleting all rows with a mean read of less than or equal to one. By using the edgeR^25^ software to further analyze the data, the differentially expressed mRNAs, lncRNAs and miRNAs were obtained. All *P* values use false discovery rate (FDR) to correct the statistical significance of the multiple test. Fold changes (log2 absolute) ≧2 and FDR adjusted to *P* < 0.01 were considered significant.

For the obtained differentially expressed mRNAs, lncRNAs, and miRNAs, we generated heat maps and volcano maps using the gplots and heatmap packages in the R platform.

### Construction of a ceRNA regulatory network

The ceRNA control network of HNSCC was mainly established by the following steps.

First, the miRcode database^26^ was used to predict interactions between lncRNA with miRNAs. miRcode provides “whole transcriptome” human microRNA target predictions based on the comprehensive GENCODE gene annotation, including >10,000 long non-coding RNA genes^26^. miRNA targets can be predicted scientifically by entering the name of the relevant lncRNA and miRNA in the miRcode website platform (http://www.mircode.org/).

Next, we used miRTarBase^15^, miRDB^16^ and TargetScan^17^ databases to retrieve miRNA targeted mRNAs. miRTarBase is a database that has accumulated more than three hundred and sixty thousand miRNA-target interactions (MTIs), which are collected by manually surveying pertinent literature and mining the text systematically to filter research articles related to functional studies of miRNAs^15^. miRDB is an online database for miRNA target prediction and functional annotations. All the targets in miRDB were predicted by mirTarget, which was developed by analyzing thousands of miRNA-target interactions from high-throughput sequencing experiments^16^. TargetScan predicts biological targets of miRNAs by searching for the presence of conserved 8mer, 7mer, and 6mer sites that match the seed region of each miRNA^17^. In order to improve the validity of our search results, we only included miRNA-targeted mRNAs present in all three databases to construct the ceRNA network.

At last, we used Cytoscape 3.5.1 software to visually map the results. Cytoscape (http://www.cytoscape.org/) is an open source software platform for visualizing molecular interaction networks and biological pathways and integrating these networks with annotations, gene expression profiles and other state data.

### Survival analysis

We use the R survival package (https://CRAN.R-project.org/package=survival, Version: 2.41-3) for survival analysis for all RNAs in the ceRNA network. The univariate Cox proportional hazards regression^27^ was carried out to identify the lncRNAs, mRNAs and miRNAs whose expression correlated with overall survival. For the overall survival rates, the log-rank test was used to compare the significant differences in univariate analysis between subgroups. Unless otherwise stated, a *P* value of less than 0.05 is considered statistically significant.

### Data availability

The datasets analysed during the current study are available in the TCGA repository, https://portal.gdc.cancer.gov/.

## Results

### Differentially expressed mRNAs (DEmRNAs) in HNSCC

**Figure 1:**
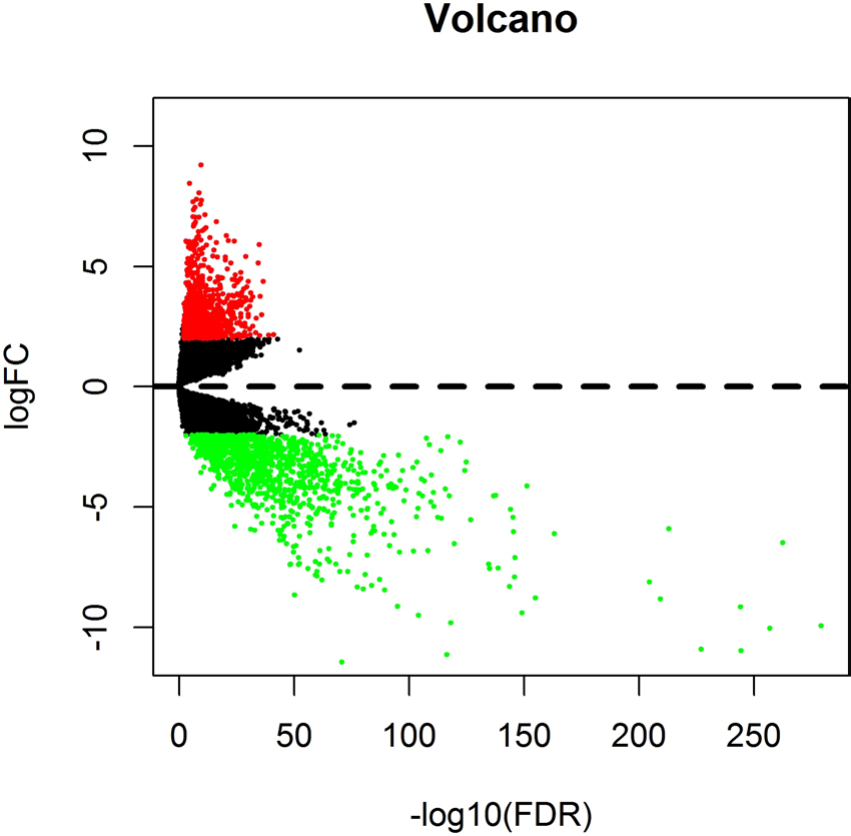
Volcano map of DEmRNAs. Red spots represent up-regulated genes, and green spots represent down regulated genes.

This study investigated the expression levels of RNAs in 502 tumor tissues (cohort T) and 44 normal tissues (cohort N). A differentially expressed gene is defined as a gene whose log2 value of the differential expression multiple value (logFC) is greater than 2 and the corrected *P* value (FDR) is less than 0.01. According to this standard, 869 (43.51%) up-regulated mRNAs and 1128 (56.49%) down–regulated mRNAs were identified by using the edgeR package. The first 25 up-regulated mRNAs, the first 25 down-regulated mRNAs, and their corresponding logFC, *P*-value, and FDR values, generated by edgeR, are outlined in Table 1. A complete list of DEmRNAs is provided in appendix 1. In Fig. 1, we show the distribution of all the differentially expressed mRNAs on the two dimensions of -log (FDR) and logFC through a volcano map. All the mRNA expression levels were standardized to the sample mean.

**Table 1:** Differentially expressed mRNAs in HNSCC samples.

Top 25 up-regulated mRNAs				Top 25 down-regulated mRNAs			
mRNA	logFC	*P* Value	FDR	mRNA	logFC	*P* Value	FDR
GPRIN1	2.157054	2.10E-43	7.51E-42	CST2	−9.93198	2.84E-284	5.13E-280
MYBL2	2.134499	3.45E-41	1.13E-39	PM20D1	−6.49148	2.74E-267	2.48E-263
IL11	4.377328	6.55E-39	2.00E-37	CA6	−10.036	1.50E-261	9.07E-258
MFAP2	2.987785	4.64E-38	1.37E-36	CST4	−10.976	9.53E-249	4.32E-245
HOXC6	3.757755	2.20E-37	6.24E-36	PRH1	−9.15828	1.91E-248	6.93E-245
HSD17B6	2.11012	3.16E-37	8.87E-36	LCN1	−10.9191	1.92E-231	5.79E-228
CA9	5.897799	5.38E-37	1.49E-35	KLK1	−5.91632	3.27E-217	8.46E-214
MMP11	5.127909	1.45E-36	3.97E-35	KRT35	−8.82614	1.40E-213	3.17E-210
GJC1	2.843289	1.06E-35	2.75E-34	THRSP	−8.12729	1.06E-208	2.13E-205
SERPINH1	2.232512	2.17E-35	5.56E-34	PRR27	−12.7635	4.92E-179	8.91E-176
HOXC9	3.726173	2.89E-34	7.01E-33	ACSM6	−6.12303	3.36E-167	5.54E-164
COL4A1	2.516886	4.39E-34	1.05E-32	HTN1	−13.2846	2.91E-161	4.39E-158
HOXA10	3.582547	3.43E-33	7.82E-32	BPIFA2	−12.9032	3.22E-159	4.49E-156
GRIN2D	3.895525	3.70E-33	8.40E-32	DGAT2L6	−8.77784	7.51E-159	9.71E-156
FZD2	2.163493	5.80E-33	1.31E-31	CCL28	−4.12258	2.93E-155	3.54E-152
DNMT3B	2.376908	6.56E-33	1.47E-31	AMY1B	−9.40408	4.93E-153	5.58E-150
ARTN	3.343319	8.38E-33	1.87E-31	NXPE4	−7.10435	6.00E-150	6.39E-147
CAMK2N2	2.894275	3.02E-32	6.60E-31	LPO	−7.91108	9.88E-150	9.94E-147
LOXL2	3.002695	3.44E-32	7.50E-31	KRT71	−6.03346	3.71E-149	3.53E-146
OFCC1	4.369364	4.20E-32	9.12E-31	PLIN1	−5.4412	4.59E-149	4.16E-146
HOXC8	4.148563	1.45E-31	3.07E-30	GPD1	−5.09309	6.00E-148	5.17E-145
DPF1	3.213548	1.94E-31	4.07E-30	AWAT2	−8.31313	2.56E-147	2.10E-144
LAMC2	3.582455	3.03E-31	6.27E-30	KRT85	−7.54774	1.90E-142	1.50E-139
SNX10	2.117463	4.01E-31	8.24E-30	OMG	−4.52724	2.16E-141	1.63E-138
COL10A1	5.402562	4.44E-31	9.09E-30	MPO	−4.54842	2.48E-140	1.79E-137

From Table 1 and Fig. 1, we can see that the difference of down-regulated mRNAs is more significant than the up-regulated mRNAs, but the logFC value between them is relatively close.

**Figure 2:**
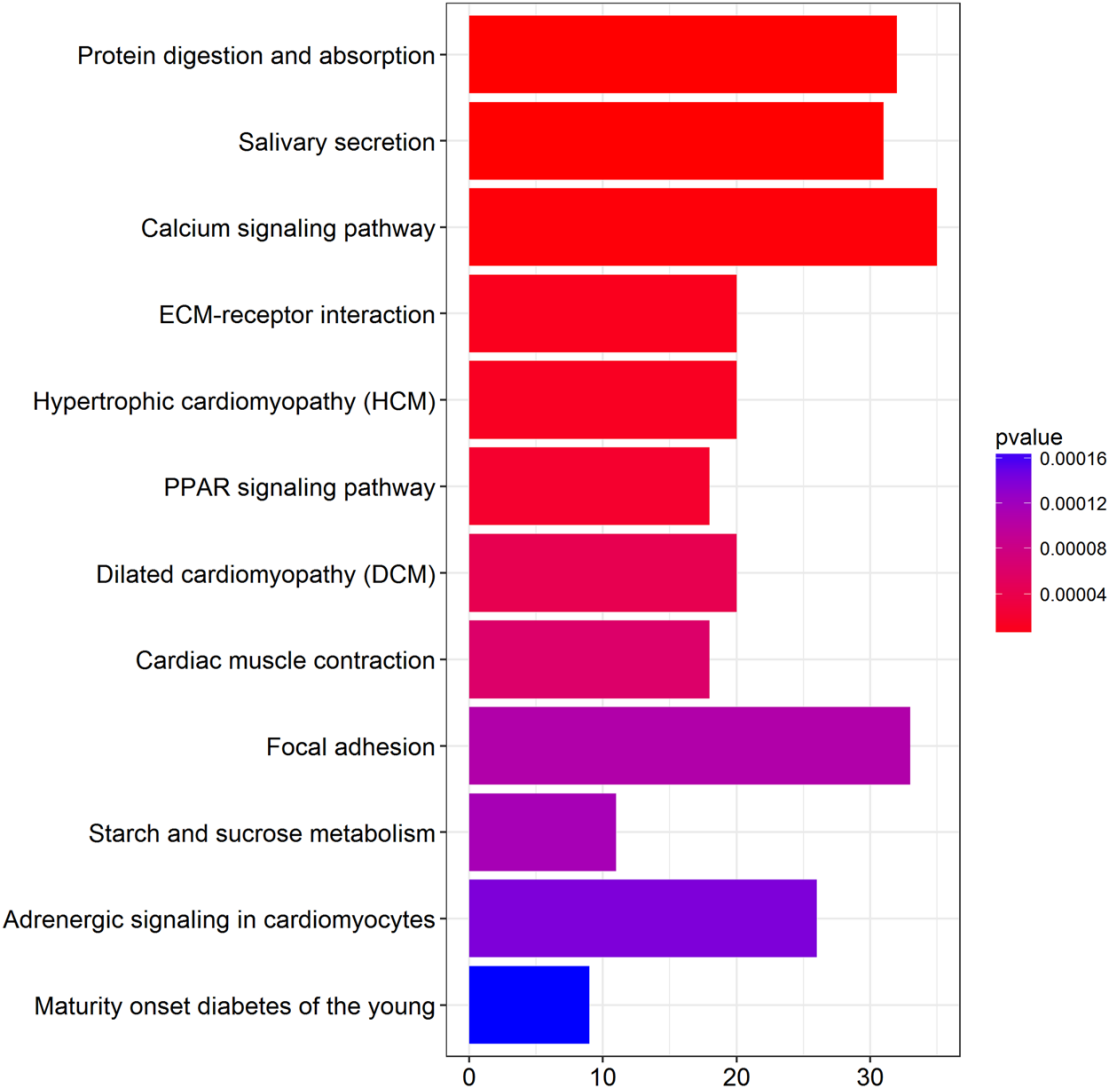
The first 12 pathways with the most significant p-values. The x-axis represents the number of DE mRNAs involved in the pathway.

Meanwhile, in order to better understand the mechanisms involved in the development of head and neck squamous cell carcinoma, we used the ClusterProfiler package in the R language for KEGG analysis and mapping, to further analyze the functional characteristics of differentially expressed RNA. The 23 significantly enriched KEGG pathways were listed in Table 2, and the first 12 pathways with the most significant p-values were shown in Fig. 2.

**Table 2:** The complete list of 23 significantly enriched KEGG pathways (pvalueCutoff = 0.05 and qvalueCutoff = 0.05).

ID	Description	*p*-value	*p*.adjust	Count
hsa04974	Protein digestion and absorption	4.04E-13	1.13E-10	32
hsa04970	Salivary secretion	2.51E-12	3.52E-10	31
hsa04020	Calcium signaling pathway	2.37E-06	0.000221	35
hsa04512	ECM-receptor interaction	9.82E-06	0.000668	20
hsa05410	Hypertrophic cardiomyopathy (HCM)	1.19E-05	0.000668	20
hsa03320	PPAR signaling pathway	1.89E-05	0.000884	18
hsa05414	Dilated cardiomyopathy (DCM)	4.24E-05	0.001697	20
hsa04260	Cardiac muscle contraction	6.02E-05	0.002106	18
hsa04510	Focal adhesion	0.000108	0.003246	33
hsa00500	Starch and sucrose metabolism	0.000116	0.003246	11
hsa04261	Adrenergic signaling in cardiomyocytes	0.000141	0.003599	26
hsa04950	Maturity onset diabetes of the young	0.000168	0.003913	9
hsa00140	Steroid hormone biosynthesis	0.000237	0.004571	14
hsa04080	Neuroactive ligand-receptor interaction	0.000243	0.004571	41
hsa00830	Retinol metabolism	0.000245	0.004571	15
hsa04971	Gastric acid secretion	0.000401	0.007012	16
hsa05202	Transcriptional misregulation in cancer	0.000793	0.013055	29
hsa04727	GABAergic synapse	0.000896	0.013615	17
hsa04972	Pancreatic secretion	0.000924	0.013615	18
hsa04371	Apelin signaling pathway	0.000976	0.013667	23
hsa04024	cAMP signaling pathway	0.001045	0.01394	30
hsa04911	Insulin secretion	0.001674	0.021304	16
hsa05412	Arrhythmogenic right ventricular cardiomyopathy (ARVC)	0.002331	0.028382	14

### Differentially expressed lncRNAs (DElncRNAs) in HNSCC

**Figure 3:**
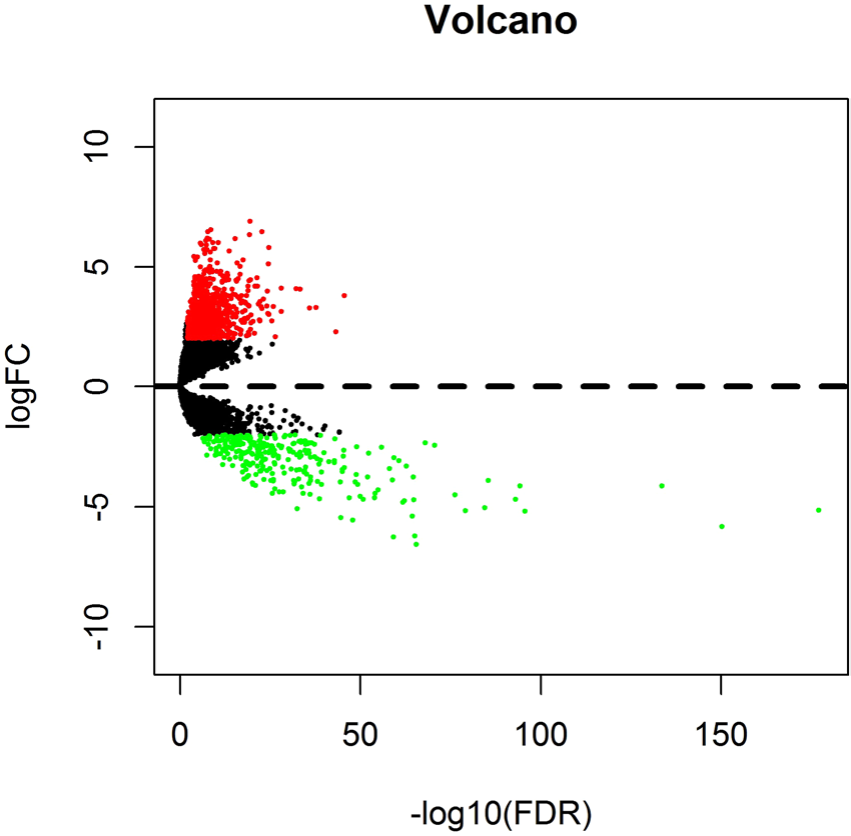
Volcano map of DElncRNAs. Red spots represent up-regulated genes, and green spots represent down regulated genes.

We identified 1041 lncRNAs to have statistically significant differential expression (logFC > 2 and FDR < 0.01)in HNSCC samples compared to normal tissues, including 755 up-regulated lncRNAs (72.5%) and 286 down-regulated lncRNAs (27.5%). The first 25 up-regulated lncRNAs and the first 25 down-regulated lncRNAs are outlined in Table 3. The distribution of all the differentially expressed lncRNA on -log (FDR) and logFC are depicted in the volcano map in Fig. 3.

**Table 3:** Differentially expressed lncRNAs in HNSCC samples.

Top 25 up-regulated lncRNAs				Top 25 down-regulated lncRNAs			
lncRNA	logFC	*P* Value	FDR	lncRNA	logFC	*P* Value	FDR
HOXC-AS2	3.7846	1.49E-48	2.85E-46	SLC8A1-AS1	−5.1342	9.90E-182	7.58E-178
FOXD2-AS1	2.2903	4.61E-46	7.22E-44	LINC01829	−5.8205	1.44E-154	5.50E-151
AC114956.1	3.2942	1.66E-40	1.93E-38	IL12A-AS1	−4.1194	9.77E-138	2.50E-134
AC114956.2	3.2753	1.14E-38	1.17E-36	AC005165.1	−5.1730	1.19E-99	2.27E-96
LINC01633	4.0588	6.75E-36	5.39E-34	AC005532.1	−4.1191	3.54E-98	5.43E-95
AC134312.5	4.0872	9.91E-35	7.10E-33	AC027130.1	−4.6787	7.70E-97	9.83E-94
LINC02081	4.1130	1.28E-30	7.31E-29	FOXCUT	−3.8970	3.74E-89	4.10E-86
RNF144A-AS1	3.1358	1.75E-30	9.88E-29	HCG22	−5.0320	3.56E-88	3.41E-85
GSEC	2.0810	8.20E-29	4.39E-27	AL357093.2	−5.1573	9.00E-83	7.66E-80
HOXA10-AS	3.3317	2.29E-28	1.16E-26	AL356123.2	−4.5085	6.59E-80	5.05E-77
U62317.2	2.7338	7.97E-28	3.75E-26	AP000866.2	−2.4229	3.38E-74	2.36E-71
DNAH17-AS1	3.0039	4.50E-27	2.00E-25	ZNF710-AS1	−2.3392	1.75E-71	1.12E-68
LINC01614	5.7936	4.89E-27	2.17E-25	AL137246.2	−6.5634	5.67E-69	3.35E-66
LINC01929	5.1264	8.05E-27	3.51E-25	LINC02303	−6.2229	1.42E-68	7.80E-66
AL358334.2	3.0447	1.14E-26	4.96E-25	LINC00443	−4.7040	3.42E-68	1.75E-65
LINC00941	3.9753	1.26E-26	5.43E-25	AL161668.3	−3.7569	4.58E-68	2.19E-65
SLC12A5-AS1	3.3598	2.13E-26	8.91E-25	MIR133A1HG	−5.3815	1.03E-67	4.62E-65
AC008011.2	3.6566	2.08E-25	8.31E-24	AC005056.1	−3.2961	4.25E-66	1.81E-63
AC010595.1	6.4630	4.72E-25	1.81E-23	AL356123.1	−4.7422	1.47E-65	5.91E-63
HAGLROS	3.2617	5.59E-25	2.12E-23	AC002546.2	−4.8176	3.72E-65	1.42E-62
AC093520.1	3.3381	1.18E-24	4.34E-23	LINC01482	−3.0702	6.25E-64	2.28E-61
TM4SF19-AS1	2.7448	1.77E-24	6.34E-23	AC091563.1	−2.9440	1.60E-62	5.58E-60
DUXAP8	3.5420	2.20E-24	7.82E-23	LINC00314	−6.2686	2.28E-62	7.61E-60
AC078778.1	2.7801	2.54E-24	8.90E-23	AP003500.1	−3.8788	4.36E-62	1.39E-59
FIRRE	3.4348	5.06E-24	1.72E-22	FALEC	−3.4100	2.89E-61	8.86E-59

As demonstrated in Table 3 and Fig. 3, most of the differentially expressed lncRNAs are up-regulated (72.5%), but the statistical significance in the down-regulated lncRNAs is more prominent.

### Differentially expressed miRNAs (DEmiRNAs) in HNSCC

**Figure 4:**
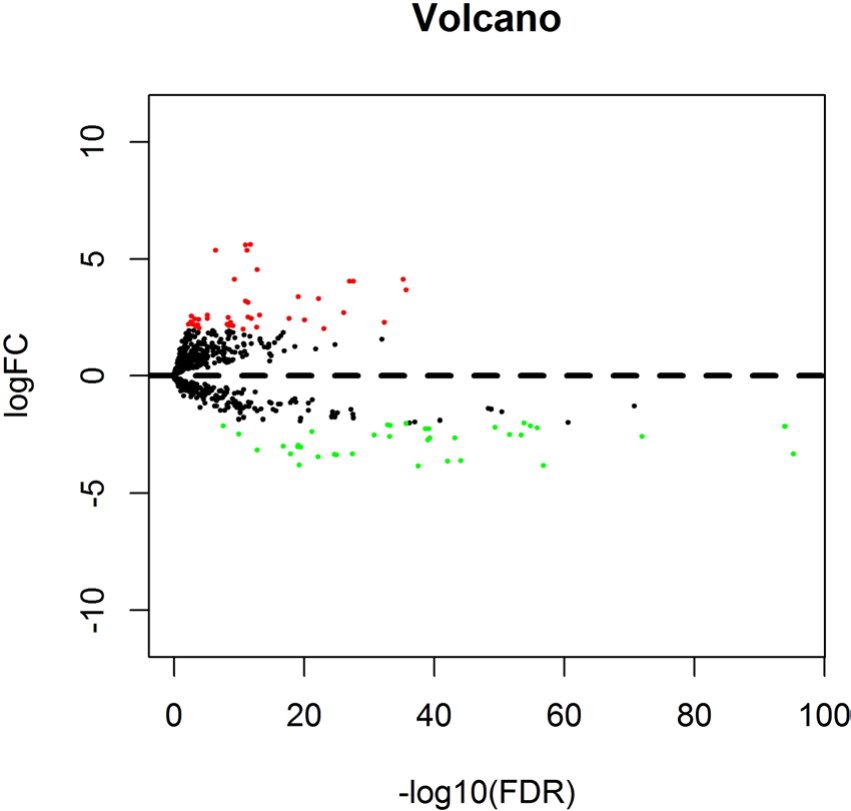
Volcano map of DEmiRNAs. Red spots represent up-regulated genes, and green spots represent down regulated genes.

To construct the lncRNA-miRNA-mRNA ceRNA regulatory network, we also compared miRNA differential expression profiles in tumor tissues and normal tissues. As a result, a total of 82 differentially expressed miRNAs were identified in samples from HNSCC patients, including 44 up-regulated miRNAs (53.6%) and 38 down-regulated miRNAs (46.4%). The first 25 up-regulated miRNAs and the first 25 down-regulated miRNAs are listed in Table, and the volcano map of the related differentially expressed miRNAs is shown in Fig. 4.

Table 4 and Fig. 4 show that, although the number of up-regulated miRNAs is slightly higher than the number of down-regulated miRNAs, the significance of the down-regulated miRNAs remains prominent.

**Table 4:** Differentially expressed miRNAs in HNSCC samples.

Top 25 up-regulated miRNAs				Top 25 down-regulated miRNAs			
miRNA	logFC	*P* Value	FDR	miRNA	logFC	*P* Value	FDR
hsa-mir-196b	3.6753	9.11E-38	2.08E-36	hsa-mir-381	−3.3291	8.07E-99	5.34E-96
hsa-mir-615	4.1339	2.64E-37	5.65E-36	hsa-mir-101-2	−2.1342	2.97E-97	9.83E-95
hsa-mir-455	2.2865	2.36E-34	4.47E-33	hsa-mir-101-1	−2.1347	5.64E-97	1.24E-94
hsa-mir-196a-2	4.0426	1.42E-29	2.48E-28	hsa-mir-378c	−2.5813	5.63E-75	9.32E-73
hsa-mir-196a-1	4.0326	6.40E-29	9.86E-28	hsa-mir-375	−3.8187	1.68E-59	1.59E-57
hsa-mir-503	2.6938	4.78E-28	7.19E-27	hsa-mir-378a	−2.2131	1.57E-58	1.30E-56
hsa-mir-301a	2.0151	6.94E-25	8.67E-24	hsa-mir-30a	−2.1199	2.01E-57	1.48E-55
hsa-mir-1910	3.2999	4.65E-24	5.71E-23	hsa-mir-195	−2.0050	2.16E-56	1.43E-54
hsa-mir-210	2.3961	7.77E-22	8.43E-21	hsa-mir-299	−2.5190	6.09E-56	3.67E-54
hsa-mir-4652	3.3788	7.79E-21	7.81E-20	hsa-mir-411	−2.4996	4.72E-54	2.60E-52
hsa-mir-301b	2.4590	2.09E-19	1.84E-18	hsa-mir-29c	−2.1922	8.91E-52	4.22E-50
hsa-mir-1293	2.6024	1.07E-14	7.01E-14	hsa-mir-885	−3.6060	2.22E-46	8.15E-45
hsa-mir-548f-1	4.5293	2.33E-14	1.51E-13	hsa-mir-378d-2	−2.6318	1.64E-45	5.70E-44
hsa-mir-937	2.0785	3.00E-14	1.89E-13	hsa-mir-378i	−3.6291	2.40E-44	7.96E-43
hsa-mir-31	2.4590	2.04E-13	1.25E-12	hsa-mir-378d-1	−2.6329	1.53E-41	4.61E-40
hsa-mir-105-1	5.6049	2.95E-13	1.76E-12	hsa-mir-486-1	−2.2494	2.17E-41	6.25E-40
hsa-mir-1305	2.5079	7.46E-13	4.29E-12	hsa-mir-4510	−2.7207	3.15E-41	8.69E-40
hsa-mir-4745	3.1281	7.60E-13	4.34E-12	hsa-mir-486-2	−2.2506	7.98E-41	2.11E-39
hsa-mir-767	5.3563	9.67E-13	5.47E-12	hsa-mir-135a-2	−3.8412	1.06E-39	2.70E-38
hsa-mir-105-2	5.5880	1.73E-12	9.60E-12	hsa-let-7c	−2.0245	9.47E-38	2.09E-36
hsa-mir-9-2	3.2009	1.80E-12	9.93E-12	hsa-mir-410	−2.1052	2.62E-35	5.41E-34
hsa-mir-9-1	3.1945	1.89E-12	1.03E-11	hsa-mir-1258	−2.5869	3.57E-35	7.16E-34
hsa-mir-9-3	3.1923	1.92E-12	1.04E-11	hsa-mir-99a	−2.0839	6.70E-35	1.30E-33
hsa-mir-1254-1	2.0027	4.73E-12	2.41E-11	hsa-mir-378f	−2.5208	8.37E-33	1.50E-31
hsa-mir-1269a	4.1252	1.16E-10	4.97E-10	hsa-mir-499a	−3.3268	2.23E-29	3.59E-28

### Construction of a ceRNA regulatory network in HNSCC

In order to better understand the role of differentially expressed lncRNAs in HNSCC and to further elucidate the interaction between these differentially expressed lncRNAs and differentially expressed miRNAs, we constructed a lncRNA-miRNA-mRNA related ceRNA regulatory network of HNSCC.

**Figure 5:**
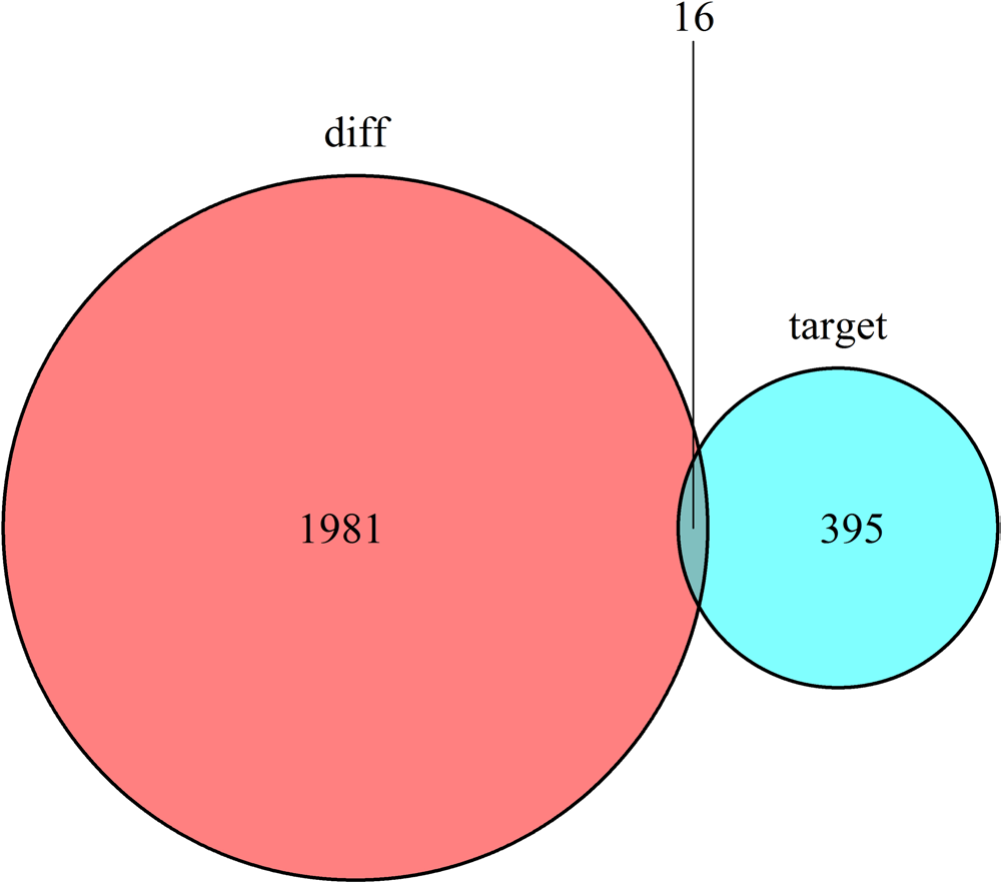
Venn diagram of mRNAs involved in ceRNA regulation network. As shown in the figure, the number of mRNA expressed in the red area is only the difference expression, rather than the target number. The blue area presents only the target number instead of the number of mRNA expressed differently, while the purple area in the middle represents the number of mRNA which is both the differential expression and the target.

**Figure 6:**
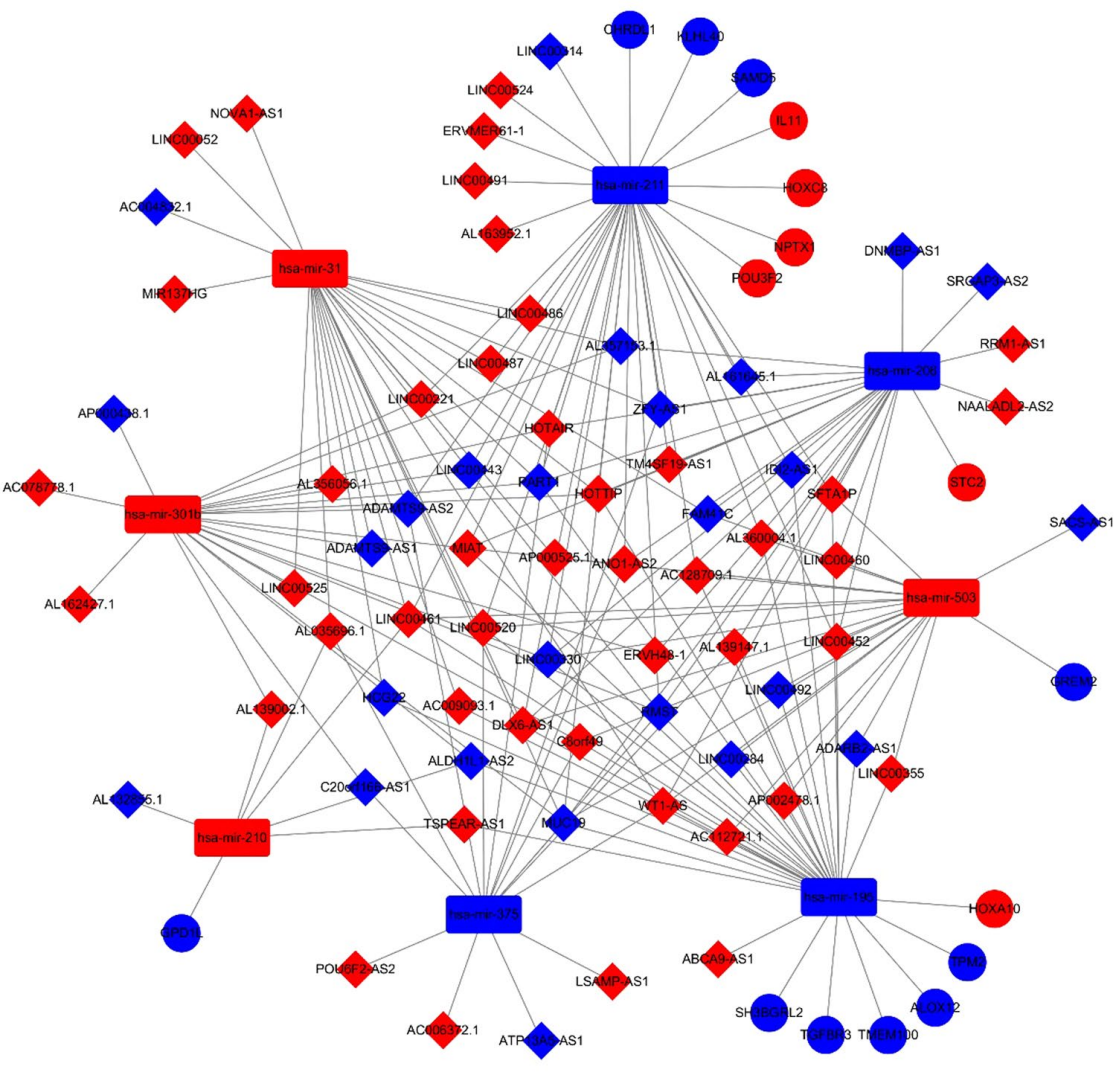
DElncRNA mediated ceRNA regulatory network in head and neck squamous cell carcinoma. The nodes highlighted in red indicate expression up-regulation, and the nodes highlighted in blue indicate expression down-regulation. IncRNAs, miRNAs and mRNAs are represented by diamonds, rounded rectangles, and ellipses, respectively.

First, we used the 1041 differentially expressed lncRNAs retrieved from the miRcode database^26^, and applied the Perl program to identify 173 pairs of interacting lncRNAs and miRNAs. From the 82 retrieved DEmiRNAs, we predicted that 8 of them could interact with 71 differentially expressed lncRNAs. Following, we found that these 8 DEmiRNAs targeted 411 mRNAs in all three databases (miRTarBase, miRDB and TargetScan). The 411 targeted mRNAs were cross-checked with the 1997 DEmiRNAs retrieved from the miRcode database. The results showed that 16 mRNAs were involved in the construction of the ceRNA regulatory network (Fig. 5). Finally, we constructed the ceRNA regulatory network of head and neck Squamous cell carcinoma by incorporating 71 DElncRNAs, 16 DEmRNAs and 8 DEmiRNAs, as shown in Fig. 6.

### Correlation analysis of survival and the expression of lncRNAs, miRNAs and mRNAs in the ceRNA network

**Figure 7:**
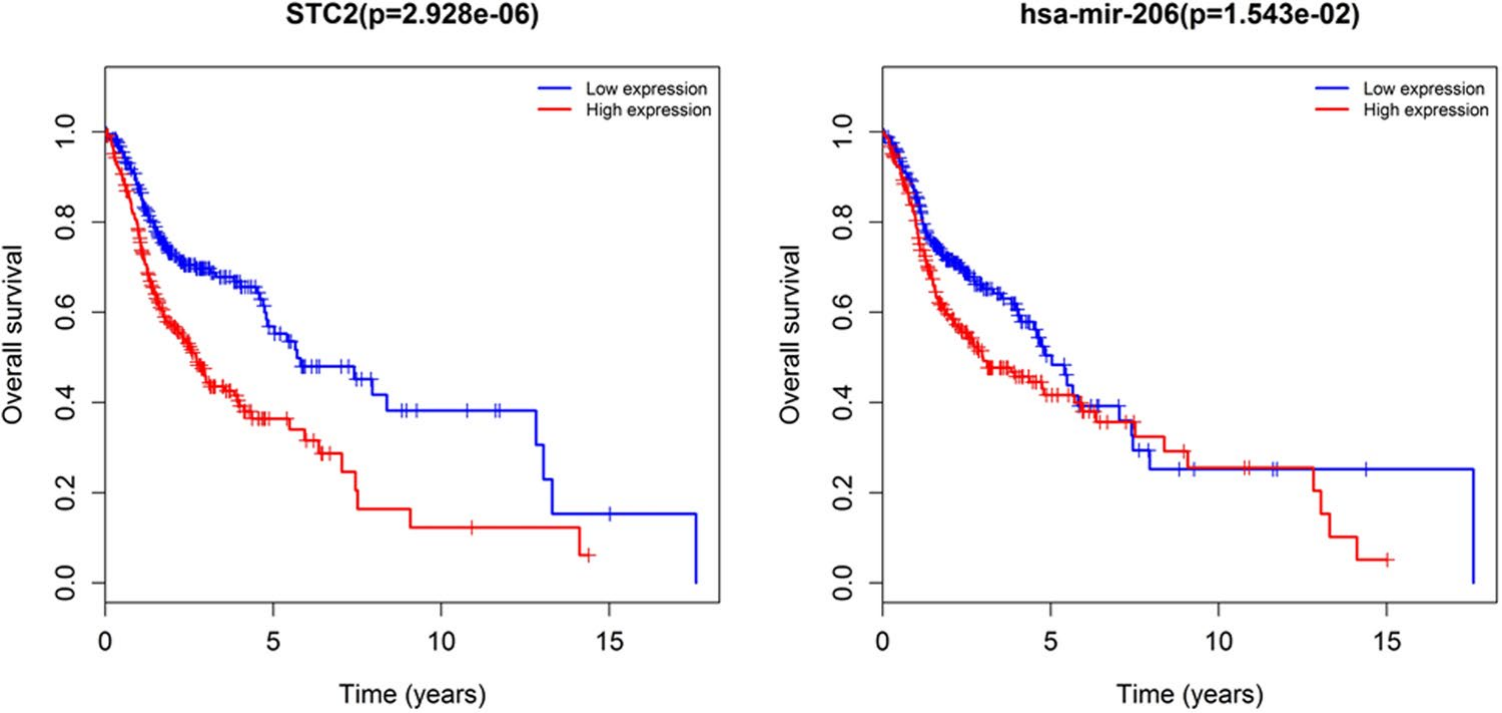
Kaplan-Meier curve analysis of DEmRNA (*STC2*), DEmiRNA (*has-mir-206*) in HNSCC patients.

**Figure 8:**
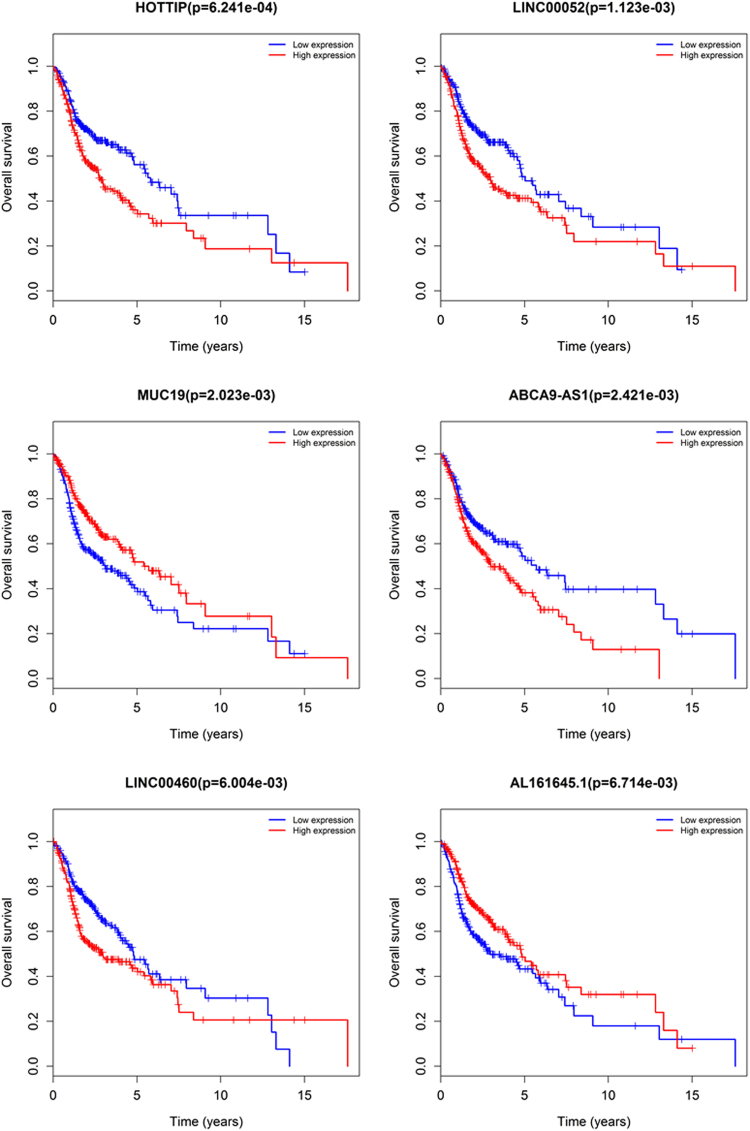
Kaplan-Meier curve analysis of DElncRNAs and overall survival rate in HNSCC patients.

**Figure 9:**
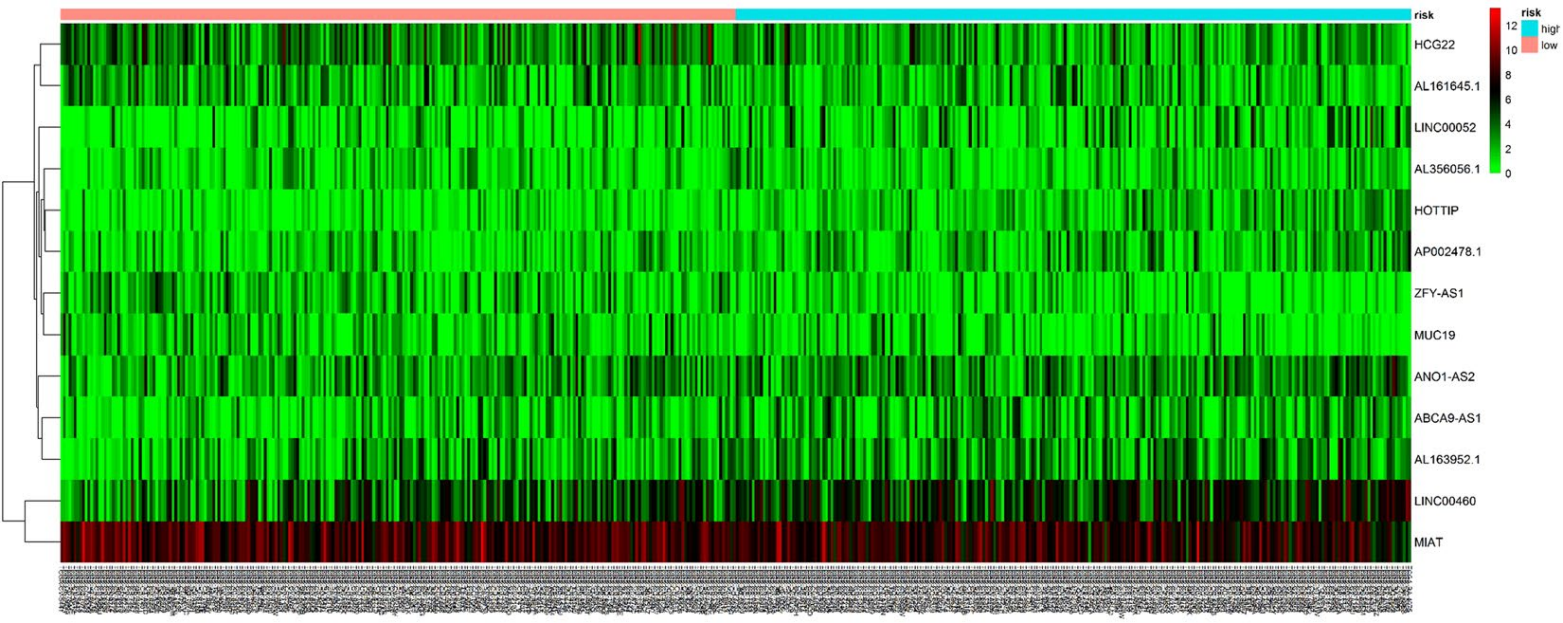
A risk heat map constructed from 13 lncRNAs from 546 HNSCC patients that have a significant impact on survival. The value of risk rises gradually from left to right.

To identify the potential lncRNAs, miRNAs and mRNAs with prognostic characteristics, the expression levels of 71 lncRNAs, 8 miRNAs and 16 mRNAs in the ceRNA network were profiled using the univariate Cox proportional hazards regression model. As a result, only one miRNA, one mRNA and thirteen lncRNAs were found to be significantly associated with overall survival (*P* < 0.05). Through the Kaplan-Meier curve analysis, we found that the expression levels of the *STC2* mRNA and *hsa-mir-206* miRNA were negatively correlated with the overall survival rate (*P* < 0.05; Fig. 7). In addition, 13 lncRNAs were closely related to survival. 8 differentially expressed IncRNA, including *ABCA9-AS1*, *AL163952*.*1*, *AL356056*.*1*, *ANO1-AS2*, *AP002478*.*1*, *HOTTIP*, LINC00052, and *LINC00460*, were negatively correlated with overall survival (*P* < 0.05). On the contrary, 5 differentially expressed IncRNA, including *AL161645*.*1*, *HCG22*, *MIAT*, *MUC19*, and *ZFY-AS1*, were positively correlated with overall survival time (*P* < 0.05). In Fig. 8, we show the Kaplan-Meier curves of 6 lncRNAs with the most significant *P* values. In the meantime, in order to better understand the relationship between the expression of these 13 lncRNAs and the patient’s survival time, we generated risk heat maps of these 13 lncRNAs in combination with clinical survival data (Fig. 9).

**Figure 10:**
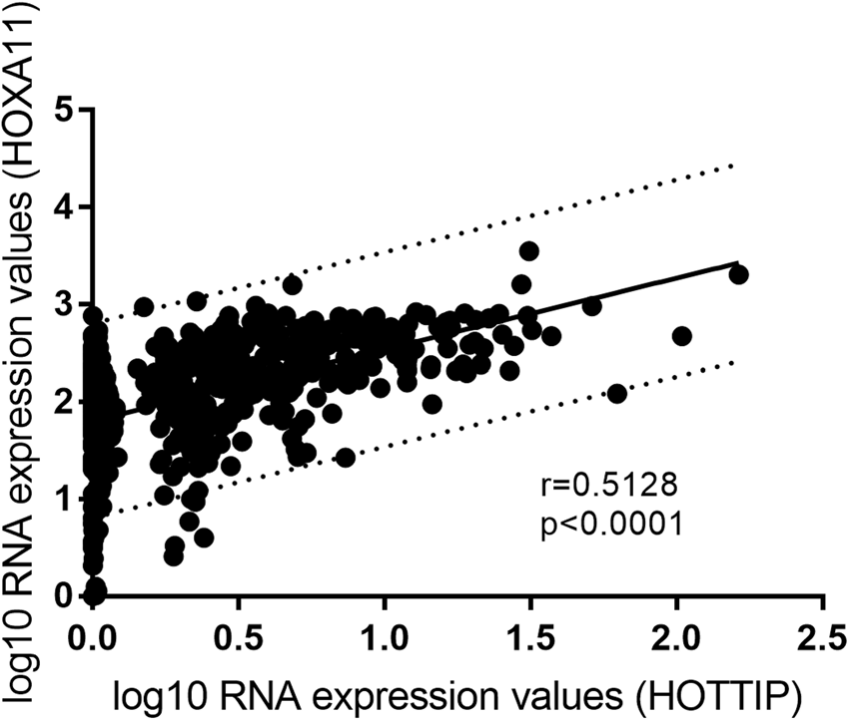
Linear regression of *HOTTIP* vs *HOXA11* expression level. Dashed lines represent 95% confidence interval.

The constructed ceRNA network showed a possible interaction between DElncRNAs and mRNAs in head and neck squamous cell carcinoma. To confirm this finding, we performed regression analysis between the expression levels of 13 DElncRNAs significantly related to survival and all 16 DEmRNAs included in the network. It was shown that most lncRNA and mRNA in the network do not have direct linear correlation, except *HOTTIP* and *HOXA10* (Fig. 10).

## Discussion

In recent years, lncRNA related research has attracted the attention of various cancer fields. Accumulative evidence shows that long-chain non-coding RNAs play a very important regulatory role in tumorigenesis and tumor progression. Although research on lncRNAs for head and neck squamous cell carcinoma is limited, a recent study has reported few differentially expressed lncRNAs such as *HOTAIR*, *NEAT1*, *UCA1*, and *MALAT1*, in oral cancers^28^. The *HOX* transcript antisense intergenic RNA (*HOTAIR*) is one of the most widely studied lncRNAs^29,30^. Different studies indicate that *HOTAIR* plays a significant role in the metastatic process and may be a predictor of poor patient prognosis when is highly expressed^31,32^. A meta-analysis, performed by Troiano *et al*., revealed that *HOTAIR*’s high expression was related to advanced tumor stage, lymph-node metastasis and poor overall survival, which demonstrated the potential prognostic role of *HOTAIR* in HNSCC^30^. Tang *et al*. first demonstrated that *HOTAIR*, *NEAT-1*, *UCA1* expression levels were up-regulated, while *MEG-3* expression levels were down-regulated in oral squamous cell carcinoma (OSCC) and its corresponding adjacent tissues^33^. They also showed that only *HOTAIR* could be detected in the patient’s saliva, especially in patients with lymph node metastases^33^. Li *et al*. found that *HOTAIR* expression in laryngeal squamous cell carcinoma (LSCC) was significantly higher than that in para-cancerous tissues, it was correlated with patients’ poor prognosis, and was an independent prognostic factor of LSCC^34^.

In the ceRNA network constructed in this paper, we found that the high expression of the lncRNA *HOTAIR* is closely related to 4 differentially expressed miRNAs (*hsa-mir-301b* is highly expressed, whilst *hsa-mir-211*, *hsa-mir-206* and *hsa-mir-375* are low expressed). The highly homologous miRNA to *hsa-mir-301b*, *hsa-miR-301a-3p*, has been proven to act as an oncogene by directly regulating the anti-oncogene *Smad4*, thereby playing a role in the emergence and development of laryngeal squamous cell carcinoma^35^. Koshizuka *et al*. showed that *miR-1* and *miR-206* were down-regulated in HNSCC clinical specimens, which is in agreement with our results. Furthermore, they found that two growth factor receptors, the epidermal growth factor receptor (EGFR) and the hepatocyte growth factor receptor (c-MET), were directly regulated by both *miR-206* and *miR-1* in HNSCC cells^36^. Bruce *et al*. has reported that metadherin was the direct target of *miR-375* and their control mechanism may represent a novel oncogenic pathway that drives human head and neck cancer (HNC) progression, possibly through the PI(3) K pathway^37^. Finally, *mir-211* has also been reported to promote head and neck carcinoma progression by targeting *TGFβRII*^38^.

Considering all the identified lncRNAs, *HOTTIP*’s abnormal expression had the most significant impact on the survival of patients (*P* = 6.241e-04). Meanwhile, the only miRNA and mRNA showing significant differences in their expression levels (*P* < 0.05) were *hsa-mir-206* and *STC2*, respectively. *HOTTIP* has been proven to be correlated with the progression and prognosis of tongue squamous cell carcinoma, pancreatic cancer, osteosarcoma, and hepatocellular carcinoma^39–42^. *miR-206* has been closely linked to the diagnosis, proliferation, and prognosis of multiple cancers, including HNSCC, by many research reports^43–46^. Yang *et al*. revealed that *STC2* controls HNSCC metastasis via the PI3K/AKT/Snail signaling pathway and that targeted therapy against *STC2* may be a novel strategy to effectively treat patients with metastatic HNSCC^47^. In 2014, Ren *et al*. demonstrated that *miR-206* was expressed at markedly low levels in a cohort of gastric tumors in comparison with their matching normal tissues, and in high amounts in gastric cancer cell lines^48^. Furthermore, they found that the *miR-206*’s anti-metastatic effect was probably mediated through targeting the metastasis regulatory gene *STC2* and other mRNAs, which were drastically down-regulated by the exogenous *miR-206*’s stable expression in SCG-7901 cells^48^. However, in our study, *STC2* was obviously down-regulated by the low expression of *mir-206* in HNSCC patients.

In addition to *HOTTIP*, we also found several lncRNAs in the ceRNA network that were closely linked to the survival of HNSCC patients. 13 lncRNAs had a significant impact on survival, and the expression of some lncRNAs showed a more obvious change trend as the risk increased. From the survival analysis, only *LINC00460* had been previously described as a therapeutic target and a novel prognostic biomarker for the diagnosis and treatment of nasopharyngeal carcinoma^49^. The remaining five lncRNAs have been identified for the first time to be closely related to the prognosis of HNSCC, which can serve as potential targets for future clinical treatments.

## Conclusion

To conclude, our study has identified differentially expressed mRNAs, lncRNAs, and miRNAs in HNSCC patients. Importantly, a ceRNA network has been constructed to propose a novel regulatory mechanism for the development of HNSCC. The lncRNAs identified in our constructed ceRNA network may have an important impact on the survival and prognosis of HNSCC patients.
